# Roxadustat promotes osteoblast differentiation and prevents estrogen deficiency-induced bone loss by stabilizing HIF-1α and activating the Wnt/β-catenin signaling pathway

**DOI:** 10.1186/s13018-022-03162-w

**Published:** 2022-05-21

**Authors:** Luyao Li, Afang Li, Li Zhu, Liangying Gan, Li Zuo

**Affiliations:** Department of Nephrology, Peking University People’s Hospital, No. 11 Xizhimen South Street, Beijing, 100044 China

**Keywords:** Roxadustat, Hypoxia-inducible factor, Osteoblast differentiation, Wnt/β-catenin pathway, Bone remodeling, Osteoporosis

## Abstract

**Background:**

Osteoporosis is a very common skeletal disorder that increases the risk of fractures. However, the treatment of osteoporosis is challenging. Hypoxia-inducible factor-1α (HIF-1α) plays an important role in bone metabolism. Roxadustat is a novel HIF stabilizer, and its effects on bone metabolism remain unknown. This study aimed to investigate the effects of roxadustat on osteoblast differentiation and bone remodeling in an ovariectomized (OVX) rat model.

**Methods:**

In vitro, primary mouse calvarial osteoblasts were treated with roxadustat. Alkaline phosphatase (ALP) activity and extracellular matrix mineralization were assessed. The mRNA and protein expression levels of osteogenic markers were detected. The effects of roxadustat on the HIF-1α and Wnt/β-catenin pathways were evaluated. Furthermore, osteoblast differentiation was assessed again after HIF-1α expression knockdown or inhibition of the Wnt/β-catenin pathway. In vivo, roxadustat was administered orally to OVX rats for 12 weeks. Then, bone histomorphometric analysis was performed. The protein expression levels of the osteogenic markers HIF-1α and β-catenin in bone tissue were detected.

**Results:**

In vitro, roxadustat significantly increased ALP staining intensity, enhanced matrix mineralization and upregulated the expression of osteogenic markers at the mRNA and protein levels in osteoblasts compared with the control group. Roxadustat activated the HIF-1α and Wnt/β-catenin pathways. HIF-1α knockdown or Wnt/β-catenin pathway inhibition significantly attenuated roxadustat-promoted osteoblast differentiation. In vivo, roxadustat administration improved bone microarchitecture deterioration and alleviated bone loss in OVX rats by promoting bone formation and inhibiting bone resorption. Roxadustat upregulated the protein expression levels of the osteogenic markers, HIF-1α and β-catenin in the bone tissue of OVX rats.

**Conclusion:**

Roxadustat promoted osteoblast differentiation and prevented bone loss in OVX rats. The use of roxadustat may be a new promising strategy to treat osteoporosis.

**Supplementary Information:**

The online version contains supplementary material available at 10.1186/s13018-022-03162-w.

## Background

Adult bone maintains its optimal quality and strength through the bone remodeling process. Bone remodeling mainly involves bone resorption by osteoclasts and bone formation by osteoblasts, which are in a dynamic balance under physiological conditions. Disruption of this balance will lead to skeletal disorders, the most representative of which is osteoporosis [1, 2]. Osteoporosis is characterized by low bone mass and deterioration of bone microarchitecture, resulting in increased susceptibility to fractures [3]. Therefore, developing effective drugs to prevent and treat osteoporosis is vital.

Hypoxia-inducible factor-1α (HIF-1α) plays an important role in bone metabolism. Previous animal studies reported that stabilization of HIF-1α via systemic administration of prolyl hydroxylase (PHD) inhibitors prevented age-related bone loss [4] and ameliorated estrogen deficiency-induced osteoporosis [5, 6]. In addition, activation of HIF-1α signaling in osteoblasts via osteoblast-specific disruption of von Hippel–Lindau protein (pVHL) strikingly protected ovariectomized mice from bone loss [7].

It is generally acknowledged that the canonical Wnt/β-catenin signaling pathway also plays a crucial role in bone metabolism [8, 9]. Activation of the Wnt/β-catenin pathway promotes osteoblast differentiation and bone formation [10, 11]. The HIF signaling pathway is closely related to the Wnt/β-catenin pathway. There have been reports that HIF-1α stabilization activates the Wnt/β-catenin signaling pathway in bone marrow mesenchymal stem cells [6] and osteoblasts [12].

Roxadustat, a novel small molecule HIF-PHD inhibitor that can stabilize HIF-α expression, is currently in clinical use for the treatment of renal anemia [13, 14]. As a potent HIF-α stabilizer, roxadustat was found to promote fracture healing by stimulating the proliferation and migration of bone marrow mesenchymal stem cells, in which activation of the HIF-1α pathway was shown to be critical [15]. Moreover, roxadustat inhibited osteoclast differentiation and reduced bone resorption in vitro [16]. However, the effects of roxadustat on osteoblast differentiation and bone remodeling remain unknown. In our study, we investigated the effect of roxadustat on osteoblast differentiation in vitro and the underlying mechanisms involving the HIF-1α and Wnt/β-catenin pathways. In addition, we explored its effect on bone remodeling using an animal model of osteoporosis.

## Materials and methods

We performed our experiments in two parts. In the first part, we used primary mouse calvarial osteoblasts to assess the in vitro effects of roxadustat on osteoblast differentiation and further explored the possible mechanisms. In the second part, we used the well-established bilateral ovariectomized (OVX) rat model to investigate the in vivo effects of roxadustat on bone remodeling.

### Primary mouse calvarial osteoblast isolation and culture

Primary calvarial osteoblasts were isolated from newborn 48-h-old C57BL/6 mice as reported previously [17]. C57BL/6 mice were purchased from Beijing Vital River Laboratory Animal Technology Co., Ltd., and were housed under pathogen-free conditions. Briefly, after removal from the skulls, the calvariae were cut into several small fragments and subjected to trypsin (Gibco, USA) digestion for 15 min. Then, the supernatant was discarded, and the remaining bone tissue was continually digested with 0.1% collagenase-II (Gibco, USA) for another 30 min at 37 ℃. The supernatant was collected, and the primary osteoblasts were cultured in basic medium (α-MEM, Gibco, USA) supplemented with 10% fetal bovine serum (FBS, Gibco, USA), 100 U/ml penicillin and 100 µg/ml streptomycin (Gibco, USA) and were maintained in a humidified, 5% CO_2_ atmosphere at 37 °C. To induce osteoblast differentiation, we used osteogenic medium containing α-MEM, 10% FBS, 100 U/ml penicillin, 100 µg/ml streptomycin, 10 mM β-glycerophosphate (Sigma-Aldrich, USA) and 50 µg/ml ascorbic acid (Sigma-Aldrich, USA). The medium was changed every 2 days.

### Cell viability assay

Cell viability was determined using CCK-8 assays. Briefly, cells were seeded at a density of 5000 cells per well in 96-well plates and incubated for 24 h. Then, roxadustat (Selleck Chemicals, Houston, TX, USA, purity = 99.95%) was added to the medium at different concentrations. After roxadustat treatment for the indicated days, 10 µl CCK-8 reagent (DOJINDO, Tokyo, Japan) was added to each well, and the cells were incubated at 37 °C for 2 h. The absorbance at 450 nm was measured with a microplate reader (Bio-Rad, Model 680, USA).

### Alkaline phosphatase (ALP) staining and Alizarin Red S staining

Cells were cultured in osteogenic medium with or without roxadustat. ALP staining was performed at day 7 of roxadustat treatment. We fixed cells with 4% paraformaldehyde and stained them using an ALP staining kit (Beyotime, China) according to the manufacturer’s protocol. Cells were stained for 15 min at room temperature, and the staining was stopped by washing with distilled water three times. The integrated staining intensity was calculated with Image-Pro Plus software (IPP, Media Cybernetics, USA).

The matrix mineralization of cells was determined by Alizarin Red S staining at day 14 of roxadustat treatment. The cells were fixed with 4% paraformaldehyde and stained with 1% Alizarin Red S (Solarbio, China), pH 4.2, for 10 min at room temperature. Then, the cells were washed with distilled water three times to eliminate nonspecific staining. To quantify extracellular matrix mineralization, the bound stain was eluted with 10% (w/v) cetylpyridinium chloride (Sigma-Aldrich, St. Louis, MO, USA) in water at room temperature for 40 min followed by dilution with the same volume of 10% (w/v) cetylpyridinium chloride [18] and then quantified by measuring absorbance at 570 nm.

### RNA extraction and quantitative real-time PCR

Total RNA was extracted from cells cultured in osteogenic medium using an RNeasy Plus kit (Qiagen, Germany), and reverse transcription was carried out with a PrimeScript RT reagent kit (Takara, Japan). Real-time PCR was performed using TB Green Premix Ex Taq II (Takara, Japan) and a CFX96 Real-Time PCR Detection System (Bio-Rad, Hercules, CA, USA). The specific primer sequences are listed in Additional file 1: Table 1. The relative mRNA expression level of target genes was normalized to β-actin expression and was calculated using the 2^−ΔΔCt^ method [19].

### Western blot analysis

Primary mouse calvarial osteoblasts and bone samples from the right femurs were lysed with RIPA lysis buffer (Beyotime, China) supplemented with protease inhibitor (Beyotime, China). The protein concentration was quantified with a BCA protein assay kit (Solarbio, China). Western blot was then performed as described previously [20]. Briefly, equal amounts of proteins from each sample were separated on a 10% SDS-PAGE gel and then transferred to PVDF membranes (Millipore, USA). The membranes were blocked with 5% nonfat milk for 1 h at room temperature and then incubated with primary antibodies against Runx2 (1:2000, abcam, ab236639), osteocalcin (OCN) (1:300, Beyotime, AF6300), HIF-1α (1:500, abcam, ab179483), β-catenin (1:500, abcam, ab68183) and β-actin (1:1000, Beyotime, AF5003) overnight at 4 °C. Then, the membranes were incubated with the appropriate secondary antibody (1:5000, Beyotime, A0208). The protein bands were visualized using Novex® Chemiluminescent HRP Substrate (Invitrogen, USA) and an enhanced chemiluminescence detection system (Pierce Biotechnology, USA). The band intensity was quantified with ImageJ software.

### Immunofluorescence

Cells were cultured in osteogenic medium with or without roxadustat (5 μM) for 4 days. After fixation, permeabilization and blocking, the cells were incubated with primary antibodies against HIF-1α (1:500, abcam, ab179483) and β-catenin (1:500, abcam, ab68183) overnight at 4 °C. Then, the cells were incubated with Alexa Fluor 594-conjugated goat anti-rabbit antibody (1:500, abcam, ab150080). Nuclei were stained with DAPI. Images were acquired with a Leica SP8 confocal microscope.

### Small interfering RNA (siRNA) transfection

HIF‐1α and negative control (NC) siRNA oligonucleotides were synthesized by Sangon Biotech (Shanghai, China). The HIF‐1α and NC siRNA sequences were as follows: HIF‐1α, (sense strand) 5’-CCAGUUACGAUUGUGAAGUUATT-3’ and (antisense strand) 5’-UAACUUCACAAUCGUAACUGGTT-3’; NC, (sense strand) 5’-UUCUCCGAACGUGUCACGUTT-3’ and (antisense strand) 5’-ACGUGACACGUUCGGAGAATT-3’. Cells were seeded in 12-well plates and cultured to 70% confluence before transfection. Cells in each well were transfected with 50 pmol siRNA added to Opti-MEM (Gibco, USA) containing Lipofectamine 3000 (Invitrogen, USA) according to the manufacturer’s protocol. The medium was changed to complete medium 6 h after cell transfection. Twenty-four hours after transfection, the cells were cultured in osteogenic medium with or without roxadustat (5 μM). The knockdown efficiency at the mRNA and protein levels was evaluated by quantitative real-time PCR and western blot analysis, respectively.

### Animal procedures

Twenty-four 12-week-old female Sprague–Dawley rats were purchased from Vital River Laboratories (Beijing, China). The rats were raised under standard specific pathogen-free (SPF) conditions (four rats per cage) at 22 °C with 60% humidity and an automatic light/dark rhythm (12:12-h light/dark cycle). The rats were fed standard rat chow and provided water ad libitum. After one week of adaptation, the rats were randomly assigned to three groups (*n* = 8 per group): the sham-operated group (Sham), ovariectomized group (OVX) and OVX rats intragastrically receiving roxadustat (10 mg/kg, every other day). One week after bilateral ovariectomy, roxadustat administration was performed for 12 weeks. The sham and OVX groups were administered with equal volumes of saline by gavage. The rats in each group were weighed weekly to adjust the dosage of roxadustat. For dynamic parameters of bone histomorphometry assessment, the rats were subcutaneously injected with tetracycline (25 mg/kg) at days 13 and 12 and calcein (10 mg/kg) at days 3 and 2 before necropsy for double fluorescence labeling in the bone tissue [21, 22]. The rats were euthanized via exsanguination from the abdominal aorta after anesthesia by inhalation of isoflurane, and blood was collected for detection of serum bone turnover markers, including bone alkaline phosphatase (BAP), OCN and tartrate-resistant acid phosphatase 5b (TRACP-5b), using enzyme-linked immunosorbent assay (ELISA) kits (Elabscience Biotechnology, China) according to the manufacturer’s instructions. The left femurs were collected for assessment of static bone histomorphometric parameters using microcomputed tomography (micro-CT), and the right femurs were collected for detection of osteogenic markers and HIF-1α and β-catenin expression at the protein level in the bone tissue. The tibiae were collected for measurement of dynamic bone histomorphometric parameters.

### Micro-CT analysis

To evaluate static parameters of bone histomorphometry, micro-CT was undertaken using an Inveon MM system (Siemens, Munich, Germany). Images were acquired at an effective pixel size of 8.89 μm, voltage of 80 kV, current of 500 μA and exposure time of 1000 ms in each of the 360 rotational steps. The parameters trabecular and cortical bone mineral density (BMD), trabecular bone volume/tissue volume (BV/TV), trabecular number (Tb.N), trabecular thickness (Tb.Th), trabecular separation (Tb.Sp), percent cortical area (%Ct.Ar) and cortical thickness (Ct.Th) were calculated according to guidelines set by the American Society for Bone and Mineral Research (ASBMR) [23, 24] using an Inveon Research Workplace (Siemens, Germany). The regions of interest for evaluating static bone histomorphometric parameters of trabecular and cortical bone were selected to be 1–2 mm and 2–3 mm distal away from the femoral growth plate, respectively.

### Dynamic trabecular bone histomorphometric analysis

Paraffin sections of decalcified left tibiae were subjected to hematoxylin–eosin (HE) staining to identify osteoblasts [22, 25] for calculation of osteoblast number/bone surface (N.Ob/BS) and osteoblast surface/bone surface (Ob.S/BS) or stained with tartrate-resistant acid phosphatase (TRAP) staining solutions to identify osteoclasts [25] for calculation of osteoclast number/bone surface (N.Oc/BS) and osteoclast surface/bone surface (Oc.S/BS) using IPP software. For mineral apposition rate (MAR) and bone formation rate (BFR) analyses, the undecalcified right tibiae were embedded in methyl methacrylate [26]. After full polymerization, the frontal sections (9 μm thick) of the proximal segments were cut using a Leica RM2255 microtome (Bannockburn, USA). The region of interest was defined between 1 and 2 mm distal to the growth plate of the proximal tibia to evaluate MAR and BFR/BS (bone surface referent) using a semiautomatic image analysis system (Bioquant, USA). The parameters were determined and evaluated according to the report of the ASBMR Histomorphometry Nomenclature Committee [23].

### Statistical analysis

The experimental data are presented as the mean ± SD (standard deviation). For each in vitro experiment, the result was determined based on three independent replicates. Statistical analysis was performed with SPSS 26.0 software (SPSS, IBM Corporation, USA). Comparisons between two groups were performed using a two independent samples, two-tailed t test. One-way analysis of variance (ANOVA) followed by Tukey’s HSD post hoc test was used for comparisons among three or more groups. A value of *P* < 0.05 was considered statistically significant.

## Results

### In vitro experiments

#### Roxadustat promoted osteoblast differentiation and extracellular matrix mineralization

**Fig. 1:**
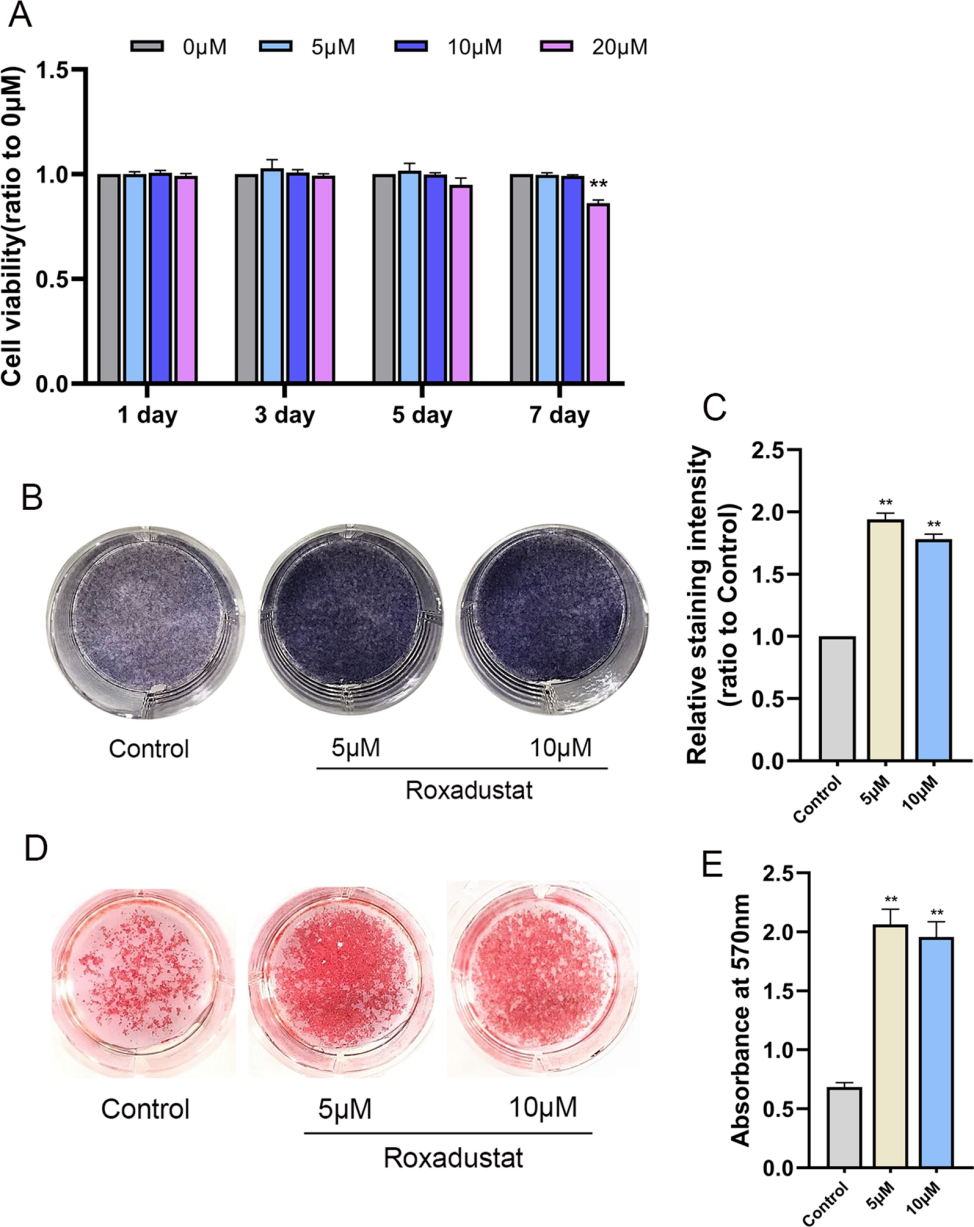
Roxadustat promoted osteoblast differentiation and extracellular matrix mineralization. **A** The viability of osteoblasts treated with roxadustat at different concentrations (0–20 μM) for 1 to 7 days, as determined by CCK-8 assays. **B**, **C** ALP staining at day 7. **D** Alizarin Red S staining at day 14. **E** Quantification of extracellular matrix mineralization. Data are presented as the mean ± SD of three independent experiments; **P < 0.01, compared with the control group (the 0 μM roxadustat treatment group)

First, we determined the effect of roxadustat on the viability of osteoblasts. We found no obvious difference in cell viability among the groups from day 1 to day 5. However, at day 7, 20 μM roxadustat significantly decreased the viability of osteoblasts (Fig. 1A). These results indicated that lower concentrations of roxadustat had no significant cytotoxicity to osteoblasts. Therefore, we chose roxadustat at concentrations of 5 μM and 10 μM for subsequent experiments.

**Fig. 2:**
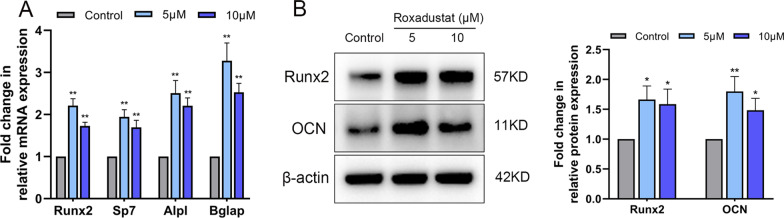
Roxadustat upregulated the expression levels of osteogenic markers in osteoblasts. **A** The mRNA expression levels of osteogenic markers in osteoblasts at day 7. **B** The protein expression levels of osteogenic markers in osteoblasts at day 7. Data are presented as the mean ± SD of three independent experiments; *P < 0.05, **P < 0.01, compared with the control group

Next, we investigated the effects of roxadustat on osteoblast differentiation. The results showed that roxadustat treatment significantly increased the ALP staining intensity in osteoblasts (Fig. 1B, C). Alizarin Red S staining showed that roxadustat significantly increased matrix mineralized nodules in osteoblasts (Fig. 1D), and the absorbance at 570 nm was remarkably higher in roxadustat-treated groups than in the control group (Fig. 1E). After roxadustat treatment for 7 days, the mRNA expression levels of Runx2 (encoding Runx2), Sp7 (encoding Osterix), Alpl (encoding ALP) and Bglap (encoding OCN) were significantly increased in roxadustat-treated cells compared with the control group (Fig. 2A). The protein expression levels of Runx2 and OCN were also obviously higher in roxadustat-treated cells than in the control group cells (Fig. 2B).

#### Roxadustat induced HIF-1α protein stabilization and subsequently activated the HIF-1α signaling pathway in osteoblasts

**Fig. 3:**
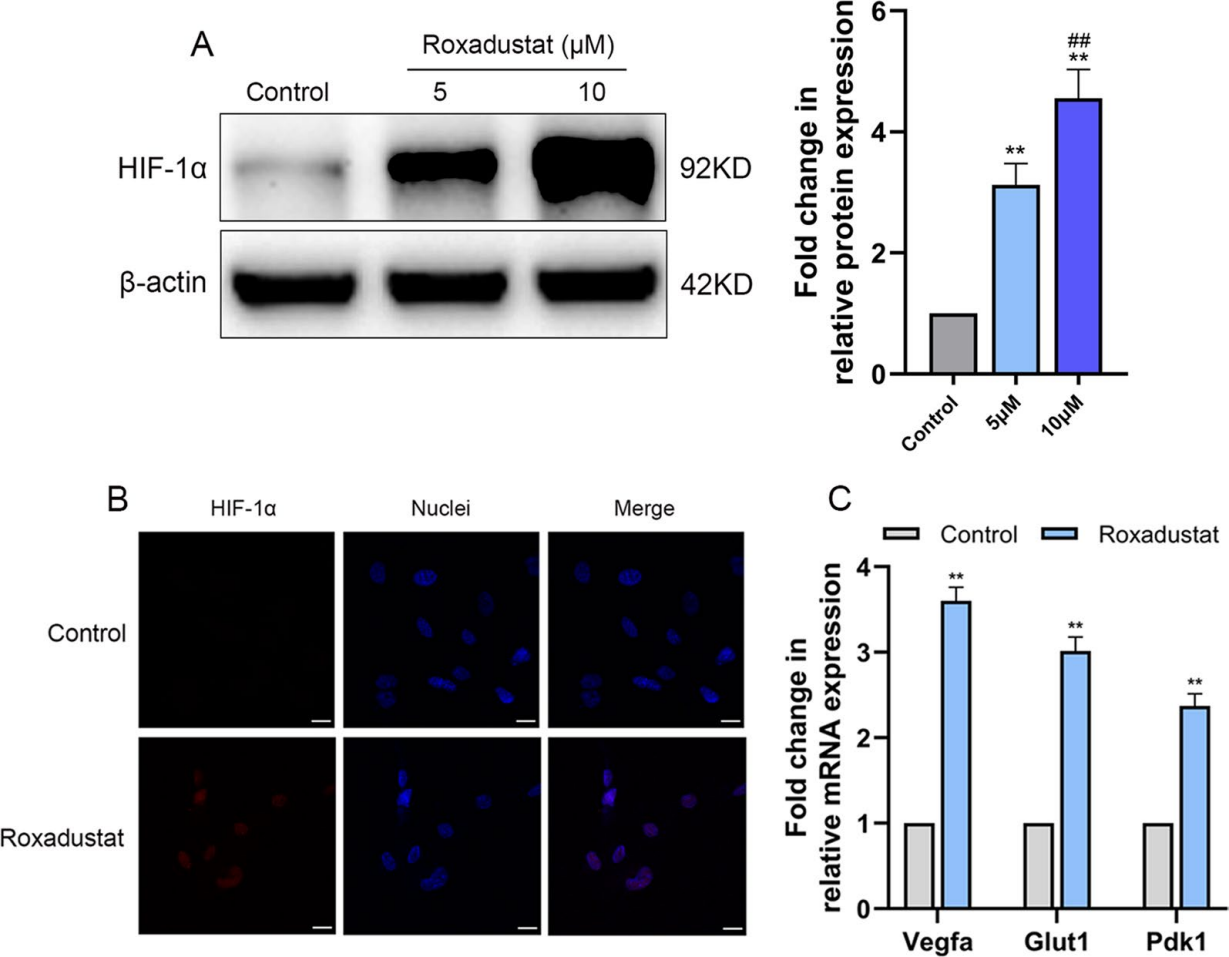
Roxadustat induced the stabilization of HIF-1α protein and activated the HIF-1α signaling pathway in osteoblasts. **A** HIF-1α protein expression in osteoblasts at day 7. **B** Representative immunofluorescence images of HIF-1α (red) and nuclear (blue) staining in the control and roxadustat-treated (5 μM) osteoblasts at day 4. **C** The mRNA expression levels of downstream HIF-1α target genes, including Vegfa, Glut1 and Pdk1 in osteoblasts at day 4. Scale bars: 20 μm. Data are presented as the mean ± SD of three independent experiments; **P < 0.01, compared with the control group; ^##^*P* < 0.01, compared with the 5 μM roxadustat treatment group

The results showed that roxadustat significantly upregulated the protein expression levels of HIF-1α in a concentration-dependent manner at day 7 (Fig. 3A). Roxadustat (5 μM) promoted nuclear accumulation of HIF-1α protein in osteoblasts (Fig. 3B) and remarkably upregulated the mRNA expression levels of downstream HIF-1α target genes, including Vegfa, Glut1 and Pdk1, compared with levels in the control group at day 4 (Fig. 3C).

#### HIF-1α stabilization played an important role in roxadustat-promoted osteoblast differentiation

To investigate whether roxadustat-promoted osteoblast differentiation was HIF-1α dependent, osteoblasts were transfected with siHIF-1α or siNC. Real-time PCR and western blot analyses showed that HIF-1α expression was successfully knocked down after transfection with siHIF-1α (See Additional file 1: Figure 1).

**Fig. 4:**
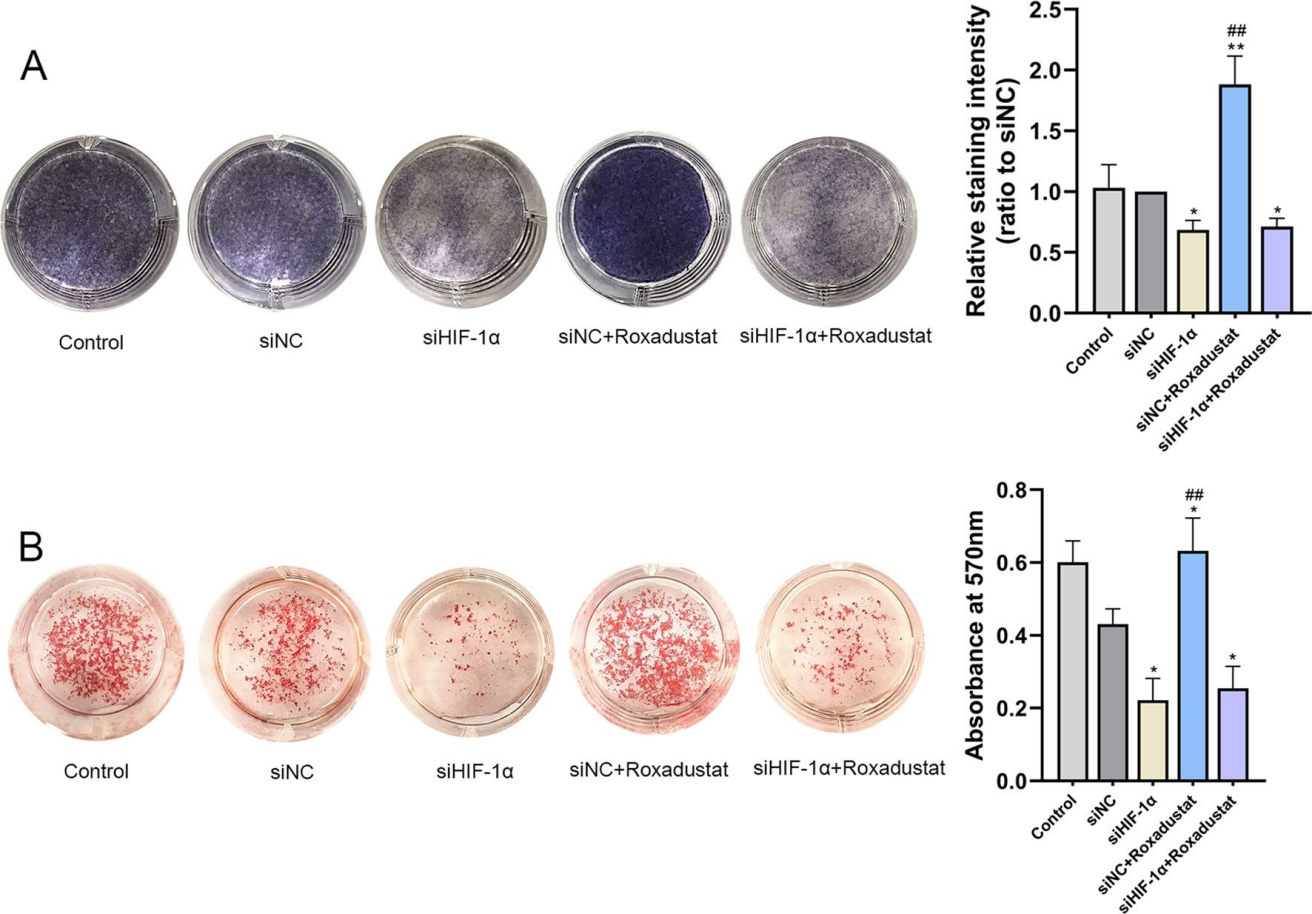
HIF-1α expression knockdown disrupted roxadustat-promoted osteoblast differentiation and matrix mineralization. **A** ALP staining at day 7. **B** Alizarin Red S staining at day 14. Data are presented as the mean ± SD of three independent experiments; *P < 0.05, **P < 0.01, compared with the siNC group; ^##^*P* < 0.01, compared with the siHIF-1α + roxadustat group. Roxadustat concentration: 5 μM

**Fig. 5:**
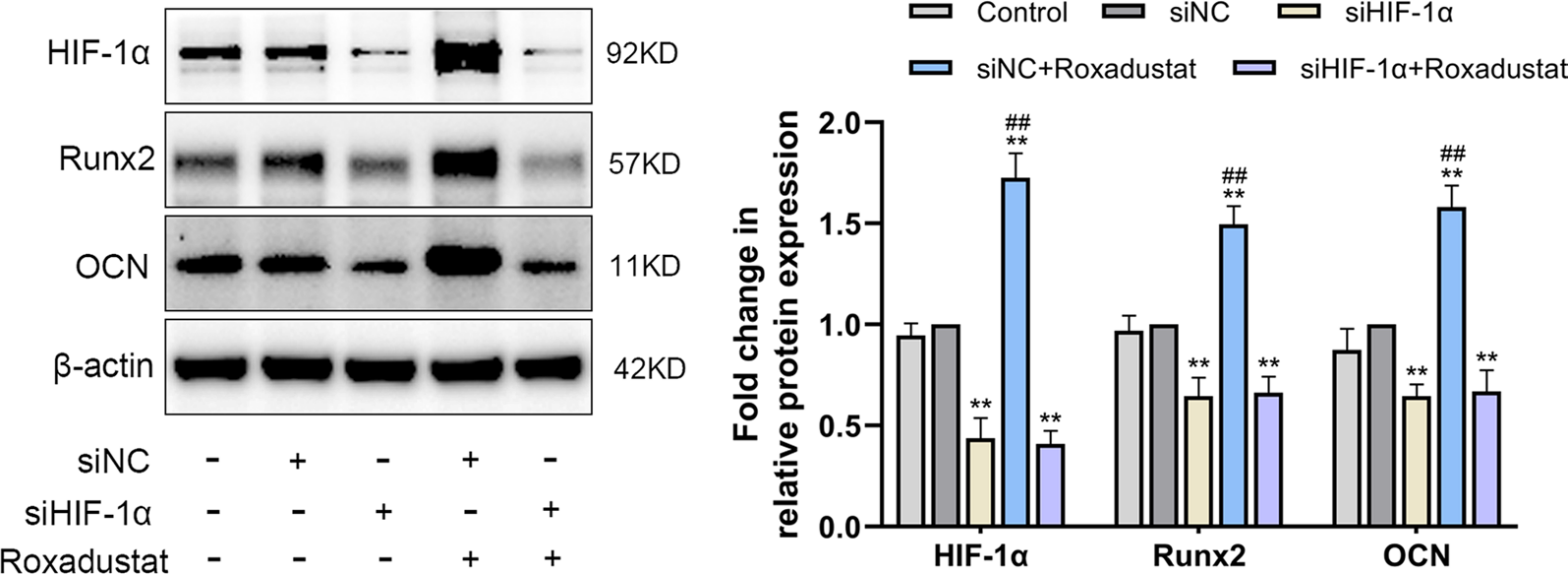
HIF-1α expression knockdown disrupted roxadustat-promoted increase in the protein expression of osteogenic markers in osteoblasts. The protein expression levels of HIF-1α, Runx2 and OCN at day 4 of roxadustat treatment. Data are presented as the mean ± SD of three independent experiments; **P < 0.01, compared with the siNC group; ^##^*P* < 0.01, compared with the siHIF-1α + roxadustat group. Roxadustat concentration: 5 μM

ALP staining and Alizarin Red S staining were performed after roxadustat treatment for 7 and 14 days, respectively. For the Alizarin Red S staining assay, at day 7, the cells were transfected with siNC or siHIF-1α for a second time to maintain HIF-1α knockdown efficiency during 14 days of roxadustat treatment. The results showed that silencing HIF-1α expression not only significantly attenuated ALP staining intensity (Fig. 4A) and reduced matrix mineralized nodule formation in osteoblasts (Fig. 4B) compared with the siNC group but also significantly attenuated roxadustat-enhanced ALP staining intensity (Fig. 4A) and matrix mineralization in the cells (Fig. 4B). In addition, HIF-1α expression deficiency significantly decreased Runx2 and OCN protein expression in the cells compared with the siNC group and abrogated the roxadustat-promoted increase in the expression of these osteogenic markers (Fig. 5).

#### Activation of the Wnt/β-catenin signaling pathway was involved in roxadustat-promoted osteoblast differentiation

**Fig. 6:**
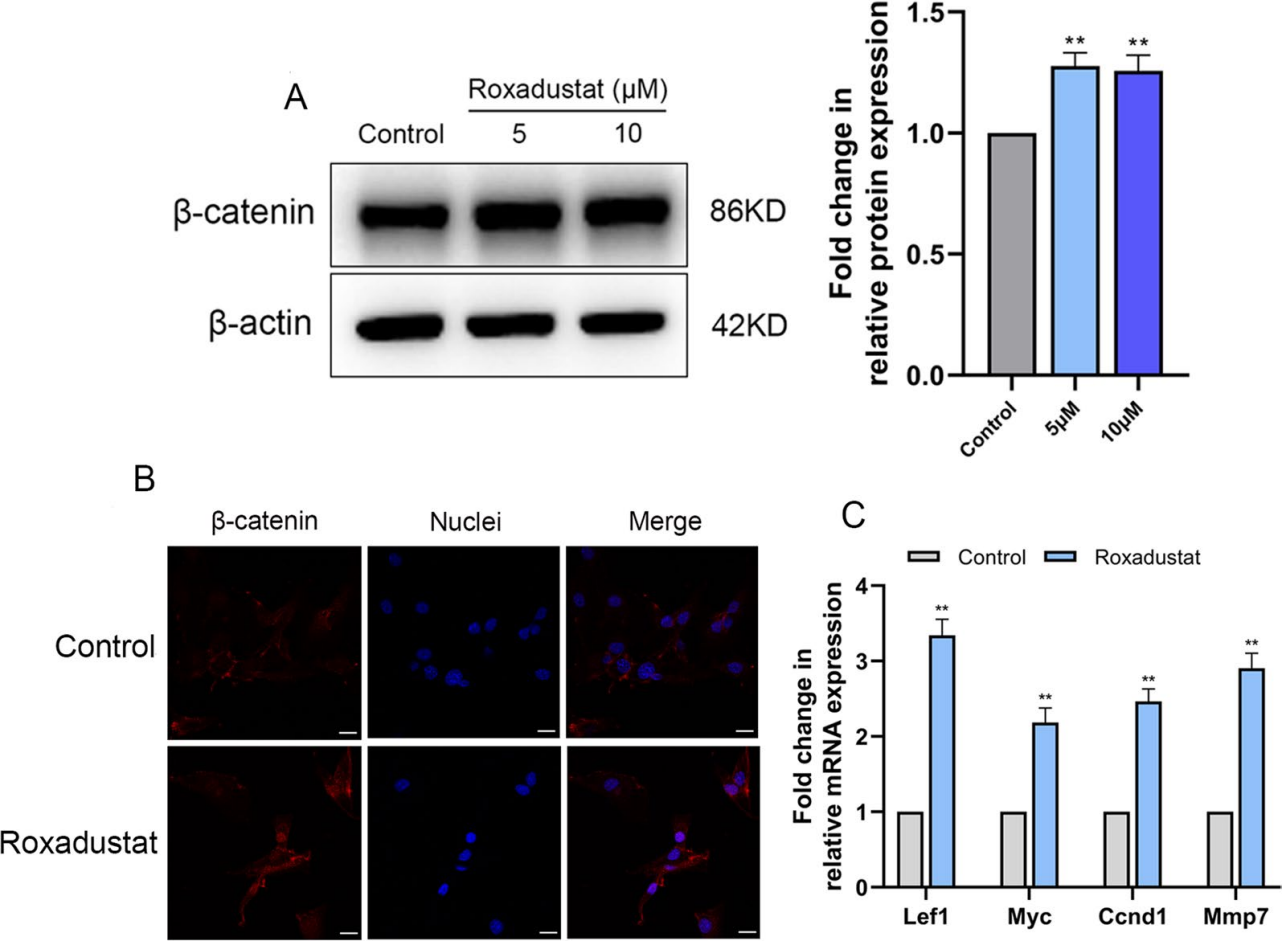
Roxadustat activated the Wnt/β-catenin signaling pathway in osteoblasts. Osteoblasts were cultured in osteogenic medium with or without roxadustat for 4 days. **A** The β-catenin protein expression level in osteoblasts. **B** Representative immunofluorescence images of β-catenin (red) and nuclear (blue) staining in the control and roxadustat-treated (5 μM) cells. **C** The mRNA expression of downstream β-catenin target genes, including Lef1, Myc, Ccnd1 and Mmp7. Scale bars: 20 μm. Data are presented as the mean ± SD of three independent experiments; **P < 0.01, compared with the control group

**Fig. 7:**
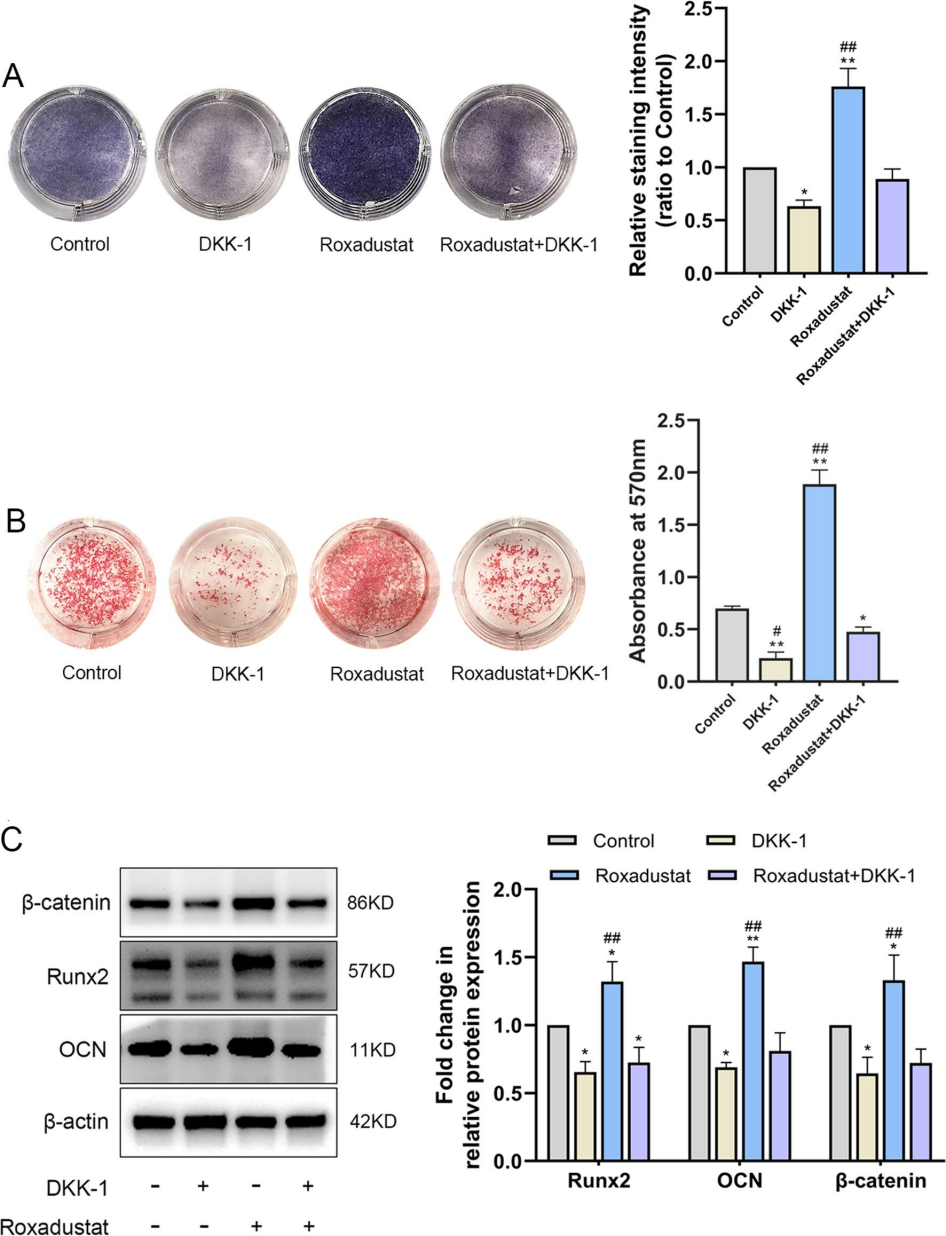
Inhibition of the Wnt/β-catenin signaling pathway attenuated roxadustat-promoted osteoblast differentiation. **A** ALP staining at day 7. **B** Alizarin Red S staining at day 14. **C** The protein expression levels of β-catenin and osteogenic markers in osteoblasts at day 4. Data are presented as the mean ± SD of three independent experiments; *P < 0.05, **P < 0.01, compared with the control group; ^#^*P* < 0.05, ^##^*P* < 0.01, compared with the Roxadustat + DKK-1 group

The results showed that roxadustat significantly increased β-catenin protein expression in osteoblasts compared with the control group (Fig. 6A). Roxadustat (5 μM) induced nuclear translocation of β-catenin protein in the cells (Fig. 6B) and obviously upregulated the mRNA expression levels of downstream β-catenin target genes, including Lef1, Myc, Ccnd1 and Mmp7 in osteoblasts compared with the control group (Fig. 6C). Furthermore, Dickkopf-1 (DKK-1) recombinant protein (500 ng/ml), which is an inhibitor of the Wnt/β-catenin signaling pathway, was added to the osteogenic medium of osteoblasts treated with or without roxadustat (5 μM). ALP staining and Alizarin Red S staining showed that DKK-1 significantly attenuated the ALP staining intensity (Fig. 7A) and matrix mineralized nodule formation in osteoblasts (Fig. 7B) compared with the control group. Meanwhile, DKK-1 also significantly attenuated roxadustat-enhanced ALP staining intensity (Fig. 7A) and matrix mineralization in the cells (Fig. 7B). In addition, DKK-1 obviously decreased β-catenin, Runx2 and OCN protein expression compared with that in the control group and abrogated the roxadustat-promoted increase in the expression of β-catenin and these osteogenic markers in the cells (Fig. 7C).

### In vivo experiments

#### Changes in body weight during the experiment and serological assessment of bone turnover markers

**Fig. 8:**
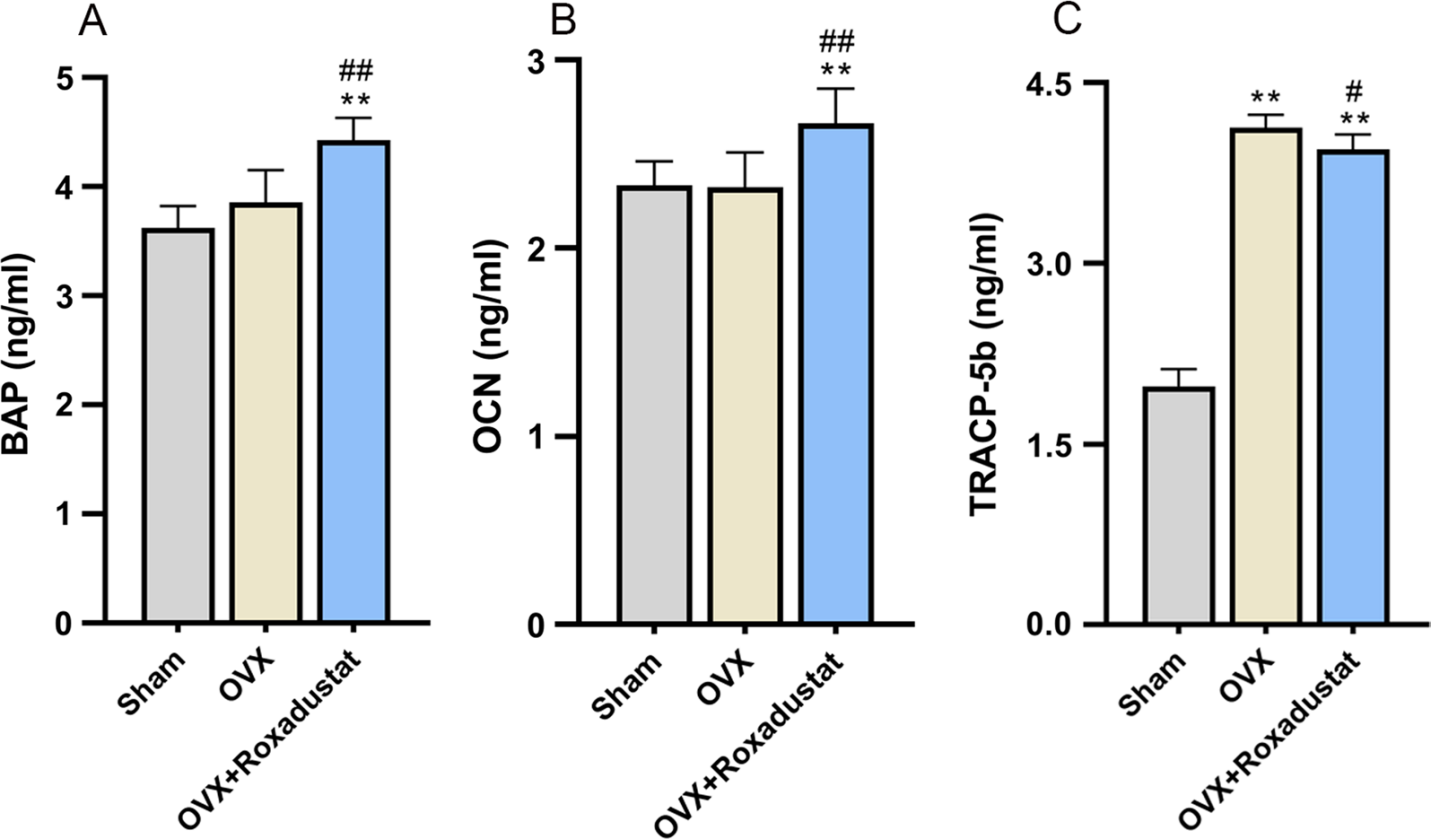
Serum levels of bone turnover markers. **A** Serum levels of BAP in the rats. **B** Serum levels of OCN in the rats. **C** Serum levels of TRACP-5b in the rats. Data are presented as the mean ± SD; **P < 0.01, compared with the sham group; ^#^*P* < 0.05, ^##^*P* < 0.01, compared with the OVX group

Throughout the entire experiment, the OVX rats gained much more weight than the sham group rats. Roxadustat administration did not affect OVX-induced body weight gain (see Additional file 1: Figure 2). After 12 weeks of roxadustat administration, the serum levels of BAP and OCN, two bone formation markers, were significantly higher in the OVX + roxadustat group than in the OVX and sham groups (Fig. 8A, B). The serum levels of TRACP-5b, a bone resorption marker, were significantly higher in OVX rats than in the sham group, but the OVX + roxadustat group had lower TRACP-5b serum levels than the OVX group (Fig. 8C).

#### Roxadustat improved OVX-induced bone loss

**Fig. 9:**
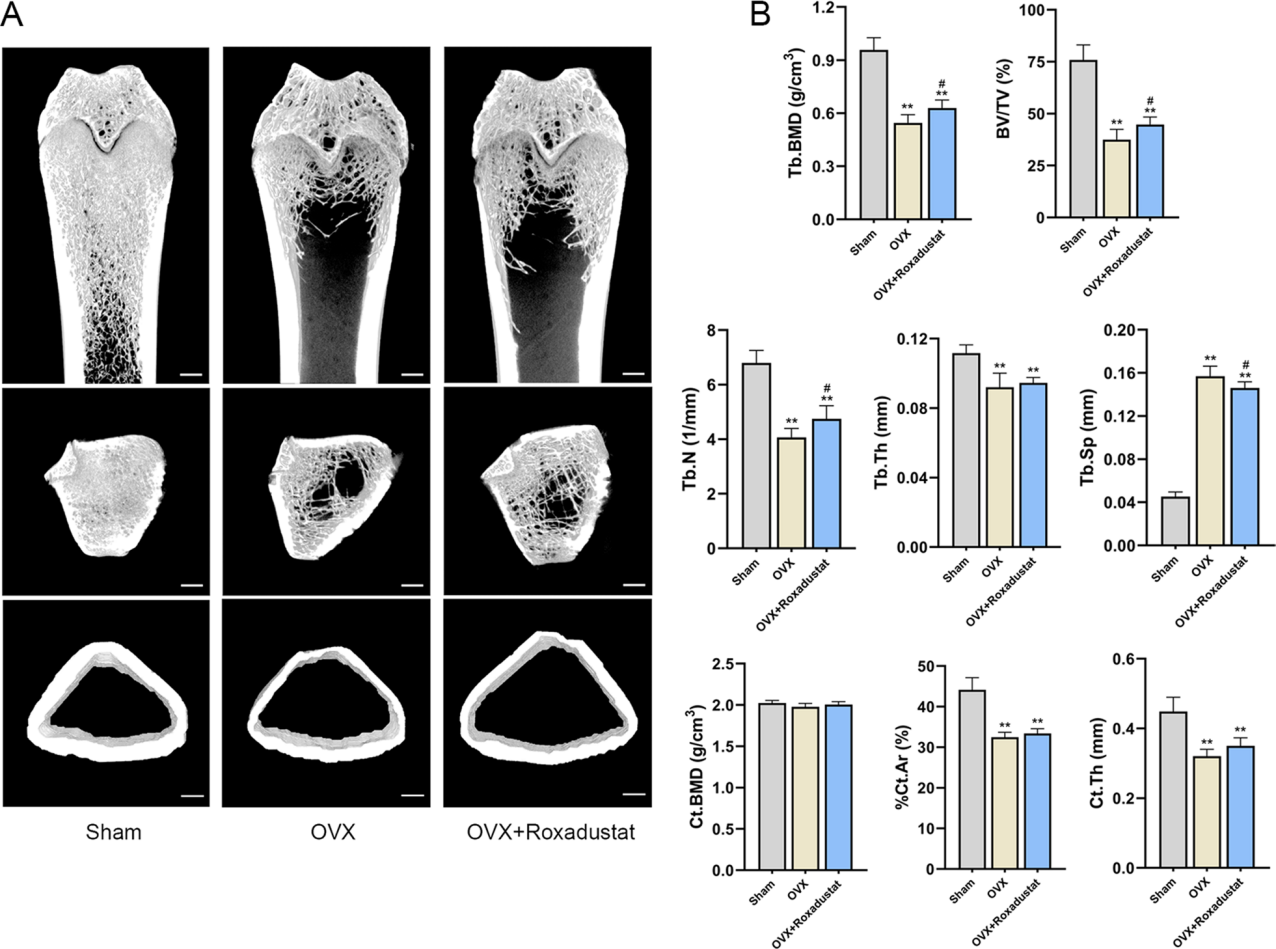
Roxadustat ameliorated OVX-induced bone loss and bone microarchitecture deterioration. **A** Representative micro-CT 3D reconstruction images of the left proximal femurs. **B** Quantitative analysis of static bone histomorphometric parameters. Data are presented as the mean ± SD; *n* = 8 per group; **P < 0.01, compared with the sham group; ^#^*P* < 0.05, compared with the OVX group. Scale bars: 1 mm. %Ct.Ar = cortical area/total tissue area

**Fig. 10:**
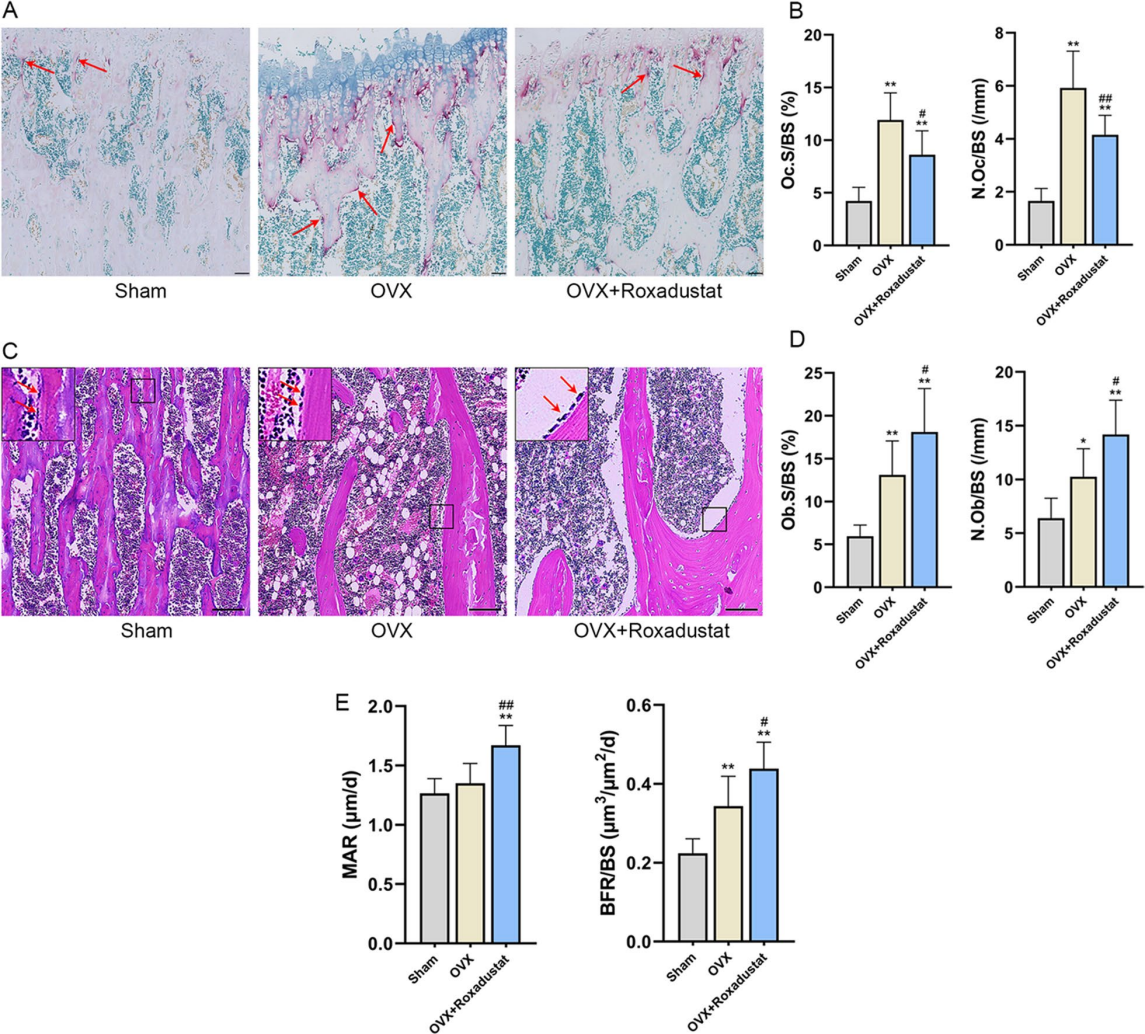
Effects of roxadustat on dynamic parameters of trabecular bone histomorphometry. **A** Representative images of TRAP staining of the left tibiae to identify osteoclasts. The arrows point to osteoclasts. Scale bars: 200 μm. **B** The calculation results for Oc.S/BS and N.Oc/BS. **C** HE staining images of the left tibiae to observe osteoblasts. The arrows point to osteoblasts. Scale bars: 100 μm. **D** The calculation results for Ob.S/BS and N.Ob/BS. **E** The calculation results for MAR and BFR/BS. Data are presented as the mean ± SD; *n* = 8 per group; *P < 0.05, **P < 0.01, compared with the sham group; ^#^*P* < 0.05, ^##^*P* < 0.01, compared with the OVX group. Oc.S/BS or Ob.S/BS: osteoclast or osteoblast surface per millimeter bone perimeter. N.Oc/BS or N.Ob/BS: osteoclast or osteoblast number per millimeter bone perimeter. MAR = double-labeled width/labeled time interval. BFR/BS = (double-labeled surface + single-labeled surface/2)*MAR/trabecular perimeter

The micro-CT results showed that the OVX rats were characterized by significantly decreased trabecular BMD, BV/TV, Tb.N, Tb.Th, %Ct.Ar, Ct.Th and dramatically increased Tb.Sp compared with the sham group. The cortical BMD showed no significant difference between OVX and sham rats. Roxadustat administration significantly increased the trabecular BMD, BV/TV and Tb.N and decreased Tb.Sp in OVX rats, while the Tb.Th, cortical BMD, %Ct.Ar and Ct.Th did not show significant difference between the OVX and OVX + roxadustat groups (Fig. 9, Table 1). In addition, the Oc.S/BS and N.Oc/BS values were significantly larger in the OVX group than in the sham group; however, these changes were notably rescued by roxadustat administration in OVX rats (Table 1, Fig. 10A, B). The OVX group had increased Ob.S/BS and N.Ob/BS compared with the sham group, and roxadustat administration further increased these parameters in OVX rats (Table 1, Fig. 10C, D). In addition, the OVX group exhibited increased BFR/BS compared with the sham group, while there was no significant difference in MAR between the two groups. Roxadustat administration obviously increased MAR and BFR/BS in OVX rats compared with the sham and OVX groups (Table 1, Fig. 10E).

**Table 1:** Effects of roxadustat on bone histomorphometric parameters

Parameters	Sham	OVX	OVX + Roxadustat
Trabecular			
BMD (g/cm^3^)	0.96 ± 0.07	0.55 ± 0.05**	0.63 ± 0.05**^#^
BV/TV (%)	75.88 ± 7.21	37.51 ± 4.93**	44.80 ± 3.61**^#^
Tb.N (1/mm)	6.79 ± 0.46	4.07 ± 0.33**	4.75 ± 0.49**^#^
Tb.Th (mm)	0.112 ± 0.005	0.092 ± 0.008**	0.095 ± 0.003**
Tb.Sp (mm)	0.045 ± 0.004	0.157 ± 0.009**	0.146 ± 0.006**^#^
Oc.S/BS (%)	4.22 ± 1.30	11.93 ± 2.55**	8.63 ± 2.27**^#^
N.Oc/BS (/mm)	1.65 ± 0.48	5.92 ± 1.39**	4.16 ± 0.73**^##^
Ob.S/BS (%)	5.95 ± 1.31	13.11 ± 3.97**	18.12 ± 5.06**^#^
N.Ob/BS (/mm)	6.40 ± 1.86	10.26 ± 2.60*	14.19 ± 3.19**^#^
MAR (μm/d)	1.27 ± 0.12	1.35 ± 0.17	1.67 ± 0.17**^##^
BFR/BS (μm^3^/μm^2^/d)	0.22 ± 0.04	0.34 ± 0.08**	0.44 ± 0.07**^#^
Cortical			
BMD (g/cm^3^)	2.02 ± 0.03	1.98 ± 0.04	2.00 ± 0.04
%Ct.Ar (%)	44.14 ± 3.02	32.46 ± 1.20**	33.38 ± 1.19**
Ct.Th (mm)	0.45 ± 0.04	0.32 ± 0.02**	0.35 ± 0.02**

Data are presented as the mean ± SD. n = 8 in each group. *P < 0.05, **P < 0.01, compared with the sham group; ^#^P < 0.05, ^##^P < 0.01, compared with the OVX group. %Ct.Ar = cortical area/total tissue area. MAR = double-labeled width/labeled time interval. BFR/BS = (double-labeled surface + single-labeled surface/2)*MAR/trabecular perimeter

#### Roxadustat administration reversed the significant OVX-induced decrease in the protein expression levels of the osteogenic markers HIF-1α and β-catenin in the bone tissue

**Fig. 11:**
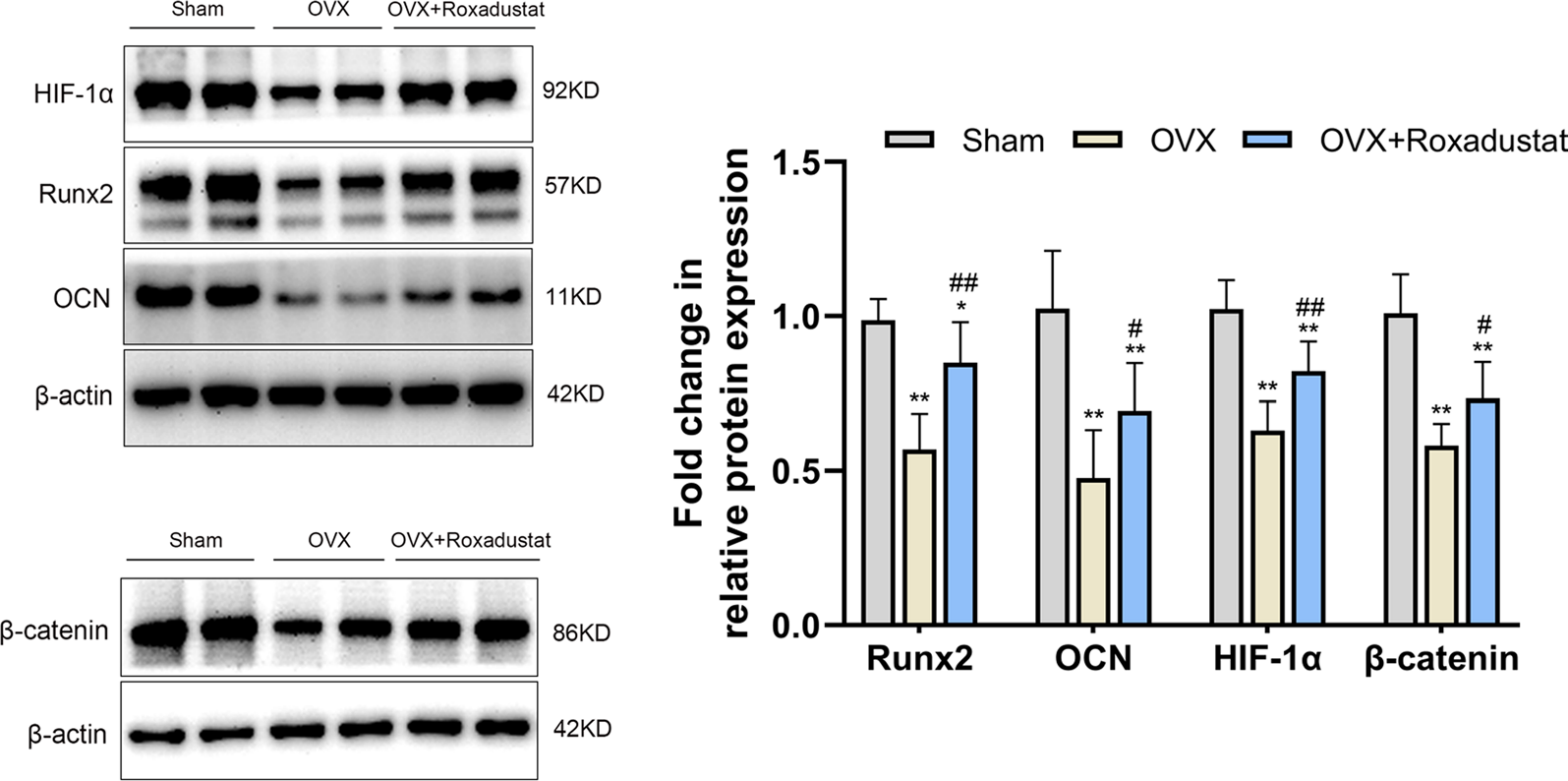
Protein expression levels of the osteogenic markers HIF-1α and β-catenin in the bone tissue. Data are presented as the mean ± SD; *P < 0.05, **P < 0.01, compared with the sham group; ^#^*P* < 0.05, ^##^*P* < 0.01, compared with the OVX group

The results showed that the protein expression levels of Runx2, OCN, HIF-1α and β-catenin were significantly decreased in the bone tissue in OVX rats compared with the sham group. However, roxadustat administration obviously increased the expression levels of these proteins in OVX rats (Fig. 11).

## Discussion

In our study, we demonstrated that roxadustat obviously promoted osteoblast differentiation in vitro through HIF-1α stabilization and activation of the Wnt/β-catenin pathway. In addition, roxadustat significantly ameliorated OVX-induced bone loss and trabecular microarchitecture deterioration by enhancing bone formation and inhibiting bone resorption.

Previous studies have suggested that genetic overexpression or pharmacological stabilization of HIF-1α protein can promote osteogenic differentiation of bone marrow mesenchymal stem cells and MC3T3-E1 cells in vitro [6, 27, 28]. In line with these studies, our study demonstrated that HIF-1α stabilization by roxadustat can also significantly promote primary osteoblast differentiation.

Our study indicated that HIF-1α stabilization acted as the key for roxadustat to promote osteoblast differentiation and that silencing HIF-1α expression abrogated roxadustat-enhanced osteoblast differentiation. In addition, we investigated whether roxadustat-promoted osteoblast differentiation was also related to the Wnt/β-catenin pathway. Activation of the Wnt/β-catenin signaling pathway, on the one hand, promotes osteoblastogenesis and bone formation and, on the other hand, inhibits osteoclastogenesis and bone resorption [29]. Several skeletal disorders, such as osteoporosis, are associated with dysregulation of this pathway [30]. Our results showed that roxadustat led to activation of the Wnt/β-catenin pathway in osteoblasts and that activation of the Wnt/β-catenin pathway was involved in roxadustat-promoted osteoblast differentiation. In addition, consistent with the in vitro results, our in vivo experiment showed that roxadustat administration significantly upregulated β-catenin protein expression in the bone tissue of OVX rats, which supported that roxadustat could also activate the Wnt/β-catenin signaling pathway in vivo. Originally, activation of HIF-1α signaling-induced coupling of angiogenesis and osteogenesis was proposed as the main mechanism responsible for the effects of HIF stabilizers on bone formation [6, 31]. Here, we investigated other possible mechanisms by which roxadustat promoted osteogenic differentiation and bone formation, laying a research foundation for further exploration of the mechanisms.

In our study, dynamic bone histomorphometric analysis and evaluation of the protein expression levels of osteogenic markers revealed that roxadustat promoted bone formation and mineralization in OVX rats. These results were consistent with our in vitro experiment regarding the effects of roxadustat on osteoblast differentiation. One study on the effects of roxadustat on osteoclasts in vitro showed that roxadustat had a direct inhibitory effect on osteoclast differentiation when CD14^+^ monocytes (preosteoblasts) were monocultured. When monocytes were cocultured with osteoblasts, inhibition of osteoclastogenesis by roxadustat was amplified, and the osteoclasts that formed in the presence of roxadustat exhibited dampened resorption activity [16]. Given the findings of this in vitro study, it might be wondered whether roxadustat could also inhibit osteoclastogenesis and bone resorption in vivo. Here, our data showed that roxadustat administration significantly decreased the Oc.S/BS and N.Oc/BS in the tibiae and lowered the serum levels of TRACP-5b in OVX rats, which confirmed that roxadustat could indeed inhibit osteoclastogenesis and bone resorption in vivo. Overall, our study is the first to clearly document that roxadustat has dual bone remodeling regulation activity: It not only promotes osteoblast-mediated bone formation but also inhibits osteoclast-dependent bone resorption in OVX rats.

In the present study, roxadustat significantly improved OVX-induced osteoporosis, which was consistent with previous studies showing that other HIF stabilizers, such as deferoxamine (DFO) and dimethyloxalylglycine (DMOG), alleviated OVX-induced osteoporosis [5, 6]. Cobalt chloride (CoCl_2_) and DFO are two representative HIF stabilizers. However, CoCl_2_ is not applied in the clinic because of its high toxicity to humans [32, 33], and DFO compromises iron metabolism [33]. Although DMOG is a promising HIF stabilizer that has been extensively studied [34, 35], it may potentially inhibit other members of the 2-oxoglutarate-dependent dioxygenase family, resulting in off-target side effects [36]. As a novel HIF stabilizer that is used for the treatment of renal anemia by promoting erythropoietin production within a physiological range, roxadustat has a short half-life [13], and in our in vivo experiment, it was administered intermittently, similar to its use in the clinic, to avoid drug-related adverse reactions induced by overexpression of downstream HIF target genes. In addition, existing clinical trials assessing the efficacy and safety of roxadustat for the treatment of anemia in patients with chronic kidney disease have indicated that roxadustat has good general safety and tolerance in clinical application [37–40]. Therefore, roxadustat may have more application advantages than other HIF stabilizers.

Of course, there are some limitations to our study. In this study, we did not perform biomechanical testing to evaluate bone strength. In addition, we did not further explore which targets of the HIF-1α and Wnt/β-catenin signaling pathways were involved in the promotive effects of roxadustat on osteoblast differentiation. These limitations should be addressed in future studies.

## Conclusion

Our study provides evidence that roxadustat can significantly promote osteoblast differentiation and improve estrogen deficiency-induced osteoporosis. Mechanistically, HIF-1 stabilization and activation of the Wnt/β-catenin signaling pathway were crucial in the effects of roxadustat. Our study suggests that roxadustat could be a potent positive regulator of bone metabolism, revealing that the use of roxadustat may serve as a new promising therapeutic strategy for skeletal disorders related to pathological bone loss such as osteoporosis.

## Supplementary Information

**Additional file 1: Table 1**. The primer sequences for real-time PCR;** Figure 1**. The knockdown efficiency of siRNA against HIF-1α at the mRNA and protein levels in osteoblasts. (A) The mRNA expression level of HIF-1α 24 h after transfection. (B) The protein expression level of HIF-1α 48 h after transfection. Data are presented as the mean ± SD of three independent experiments; **P < 0.01, compared with the siNC group; **Figure 2**. Body weight changes during the 13-week experimental period in the sham, OVX and OVX + Roxadustat groups. From week 3 to week 13, for each time point, *P* < 0.01 among the three groups in the analysis of variance for repeated measurements, and *P* < 0.01 between the OVX group (or OVX + Roxadustat group) and the sham group in the following multiple comparisons among the three groups. Week 0: the start time of ovariectomy; Week 1: the start time of roxadustat administration.

## Data Availability

All the data and materials in our study are available from the corresponding author on reasonable request.

## Abbreviations

HIF: Hypoxia-inducible factor
OVX: Ovariectomized
ALP: Alkaline phosphatase
PHD: Prolyl hydroxylase
OCN: Osteocalcin
siRNA: Small interfering RNA
BAP: Bone alkaline phosphatase
TRACP-5b: Tartrate-resistant acid phosphatase 5b
BMD: Bone mineral density
BV/TV: Trabecular bone volume/tissue volume
Tb.N: Trabecular number
Tb.Th: Trabecular thickness
Tb.Sp: Trabecular separation
%Ct.Ar: Percent cortical area
Ct.Th: Cortical thickness
N.Ob/BS: Osteoblast number/bone surface
Ob.S/BS: Osteoblast surface/bone surface
N.Oc/BS: Osteoclast number/bone surface
Oc.S/BS: Osteoclast surface/bone surface
MAR: Mineral apposition rate
BFR/BS: Bone formation rate/bone surface
SD: Standard deviation
ANOVA: One-way analysis of variance
DKK-1: Dickkopf-1

## References

[1] Lee EJ, Jain M, Alimperti S (2021). Bone microvasculature: stimulus for tissue function and regeneration. Tissue Eng Part B Rev.

[2] Kenkre JS, Bassett J (2018). The bone remodelling cycle. Ann Clin Biochem.

[3] van der Burgh AC, de Keyser CE, Zillikens MC, Stricker BH (2021). The effects of osteoporotic and non-osteoporotic medications on fracture risk and bone mineral density. Drugs.

[4] Yi L, Ju Y, He Y, Yin X, Xu Y, Weng T (2021). Intraperitoneal injection of Desferal(R) alleviated the age-related bone loss and senescence of bone marrow stromal cells in rats. Stem Cell Res Ther.

[5] Liu X, Tu Y, Zhang L, Qi J, Ma T, Deng L (2014). Prolyl hydroxylase inhibitors protect from the bone loss in ovariectomy rats by increasing bone vascularity. Cell Biochem Biophys.

[6] Peng J, Lai ZG, Fang ZL, Xing S, Hui K, Hao C (2014). Dimethyloxalylglycine prevents bone loss in ovariectomized C57BL/6J mice through enhanced angiogenesis and osteogenesis. PLoS ONE.

[7] Zhao Q, Shen X, Zhang W, Zhu G, Qi J, Deng L (2012). Mice with increased angiogenesis and osteogenesis due to conditional activation of HIF pathway in osteoblasts are protected from ovariectomy induced bone loss. Bone.

[8] Huang P, Yan R, Zhang X, Wang L, Ke X, Qu Y (2019). Activating Wnt/beta-catenin signaling pathway for disease therapy: Challenges and opportunities. Pharmacol Ther.

[9] Amjadi-Moheb F, Akhavan-Niaki H. Wnt signaling pathway in osteoporosis: epigenetic regulation, interaction with other signaling pathways, and therapeutic promises. J Cell Physiol. 2019. 10.1002/jcp.28207 10.1002/jcp.28207 30693508

[10] Yang YY, Zhou YM, Xu JZ, Sun LH, Tao B, Wang WQ (2021). Lgr4 promotes aerobic glycolysis and differentiation in osteoblasts via the canonical Wnt/beta-catenin pathway. J Bone Miner Res.

[11] Karner CM, Long F (2017). Wnt signaling and cellular metabolism in osteoblasts. Cell Mol Life Sci.

[12] Genetos DC, Toupadakis CA, Raheja LF, Wong A, Papanicolaou SE, Fyhrie DP (2010). Hypoxia decreases sclerostin expression and increases Wnt signaling in osteoblasts. J Cell Biochem.

[13] Dhillon S (2019). Roxadustat: first global approval. Drugs.

[14] Joharapurkar AA, Pandya VB, Patel VJ, Desai RC, Jain MR (2018). Prolyl hydroxylase inhibitors: a breakthrough in the therapy of anemia associated with chronic diseases. J Med Chem.

[15] Chen C, Yan S, Qiu S, Geng Z, Wang Z (2021). HIF/Ca(2+)/NO/ROS is critical in roxadustat treating bone fracture by stimulating the proliferation and migration of BMSCs. Life Sci.

[16] Hulley PA, Papadimitriou-Olivgeri I, Knowles HJ (2020). Osteoblast-osteoclast coculture amplifies inhibitory effects of FG-4592 on human osteoclastogenesis and reduces bone resorption. JBMR Plus.

[17] Taylor SE, Shah M, Orriss IR (2014). Generation of rodent and human osteoblasts. Bonekey Rep.

[18] Hao Q, Liu Z, Lu L, Zhang L, Zuo L (2020). Both JNK1 and JNK2 are indispensable for sensitized extracellular matrix mineralization in IKKbeta-deficient osteoblasts. Front Endocrinol (Lausanne).

[19] Xu G (2018). HIF-1-mediated expression of Foxo1 serves an important role in the proliferation and apoptosis of osteoblasts derived from children's iliac cancellous bone. Mol Med Rep.

[20] Yang B, Li S, Chen Z, Feng F, He L, Liu B (2020). Amyloid beta peptide promotes bone formation by regulating Wnt/beta-catenin signaling and the OPG/RANKL/RANK system. FASEB J.

[21] Meng Y, Zhang H, Li Y, Li Q, Zuo L (2014). Effects of unfractionated heparin on renal osteodystrophy and vascular calcification in chronic kidney disease rats. Bone.

[22] Chu ZM, Li HB, Sun SX, Jiang YC, Wang B, Dong YF (2017). Melatonin promotes osteoblast differentiation of bone marrow mesenchymal stem cells in aged rats. Eur Rev Med Pharmacol Sci.

[23] Dempster DW, Compston JE, Drezner MK, Glorieux FH, Kanis JA, Malluche H (2013). Standardized nomenclature, symbols, and units for bone histomorphometry: a 2012 update of the report of the ASBMR Histomorphometry Nomenclature Committee. J Bone Miner Res.

[24] Bouxsein ML, Boyd SK, Christiansen BA, Guldberg RE, Jepsen KJ, Muller R (2010). Guidelines for assessment of bone microstructure in rodents using micro-computed tomography. J Bone Miner Res.

[25] Zhou YM, Yang YY, Jing YX, Yuan TJ, Sun LH, Tao B (2020). BMP9 reduces bone loss in ovariectomized mice by dual regulation of bone remodeling. J Bone Miner Res.

[26] Erben R (1997). Embedding of bone samples in methylmethacrylate: an improved method suitable for bone histomorphometry, histochemistry, and immunohistochemistry. J Histochem Cytochem.

[27] Ying C, Wang R, Wang Z, Tao J, Yin W, Zhang J (2020). BMSC-exosomes carry mutant HIF-1alpha for Improving angiogenesis and osteogenesis in critical-sized calvarial defects. Front Bioeng Biotechnol.

[28] Xu WN, Zheng HL, Yang RZ, Jiang LS, Jiang SD (2019). HIF-1alpha regulates glucocorticoid-induced osteoporosis through PDK1/AKT/mTOR signaling pathway. Front Endocrinol (Lausanne).

[29] Moorer MC, Riddle RC (2018). Regulation of osteoblast metabolism by Wnt signaling. Endocrinol Metab (Seoul).

[30] Canalis E (2013). Wnt signalling in osteoporosis: mechanisms and novel therapeutic approaches. Nat Rev Endocrinol.

[31] Marenzana M, Arnett TR (2013). The key role of the blood supply to bone. Bone Res.

[32] Xie H, Smith LJ, Holmes AL, Zheng T, Pierce Wise J (2016). The cytotoxicity and genotoxicity of soluble and particulate cobalt in human lung epithelial cells. Environ Mol Mutagen.

[33] Zhou M, Hou J, Li Y, Mou S, Wang Z, Horch RE (2019). The pro-angiogenic role of hypoxia inducible factor stabilizer FG-4592 and its application in an in vivo tissue engineering chamber model. Sci Rep.

[34] Yuan Q, Bleiziffer O, Boos AM, Sun J, Brandl A, Beier JP (2014). PHDs inhibitor DMOG promotes the vascularization process in the AV loop by HIF-1a up-regulation and the preliminary discussion on its kinetics in rat. BMC Biotechnol.

[35] Zhang J, Guan J, Qi X, Ding H, Yuan H, Xie Z (2016). Dimethyloxaloylglycine promotes the angiogenic activity of mesenchymal stem cells derived from iPSCs via activation of the PI3K/Akt pathway for bone regeneration. Int J Biol Sci.

[36] Chan MC, Holt-Martyn JP, Schofield CJ, Ratcliffe PJ (2016). Pharmacological targeting of the HIF hydroxylases—a new field in medicine development. Mol Aspects Med.

[37] Provenzano R, Besarab A, Sun CH, Diamond SA, Durham JH, Cangiano JL (2016). Oral hypoxia-inducible factor prolyl hydroxylase inhibitor roxadustat (FG-4592) for the treatment of anemia in patients with CKD. Clin J Am Soc Nephrol.

[38] Chen N, Hao C, Liu BC, Lin H, Wang C, Xing C (2019). Roxadustat treatment for anemia in patients undergoing long-term dialysis. N Engl J Med.

[39] Provenzano R, Szczech L, Leong R, Saikali KG, Zhong M, Lee TT (2021). Efficacy and cardiovascular safety of roxadustat for treatment of anemia in patients with non-dialysis-dependent CKD: pooled results of three randomized clinical trials. Clin J Am Soc Nephrol.

[40] Fishbane S, El-Shahawy MA, Pecoits-Filho R, Van BP, Houser MT, Frison L (2021). Roxadustat for treating anemia in patients with CKD not on dialysis: results from a randomized phase 3 study. J Am Soc Nephrol.

