# Supplementary material for: Roxadustat promotes osteoblast differentiation and prevents estrogen deficiency-induced bone loss by stabilizing HIF-1α and activating the Wnt/β-catenin signaling pathway
Source: J Orthop Surg Res. 2022 May 21;17:286. doi: 10.1186/s13018-022-03162-w (PMC9124388; doi:10.1186/s13018-022-03162-w)
Supplement: Supplementary file 1 — Additional file 1: Table 1. The primer sequences for real-time PCR; Figure 1. The knockdown efficiency of siRNA against HIF-1α at the mRNA and protein levels in osteoblasts. (A) The mRNA expression level of HIF-1α 24 h after transfection. (B) The protein expression level of HIF-1α 48 h after transfection. Data are presented as the mean ± SD of three independent experiments; **P < 0.01, compared with the siNC group; Figure 2. Body weight changes during the 13-week experimental period in the sham, OVX and OVX + Roxadustat groups. From week 3 to week 13, for each time point, P < 0.01 among the three groups in the analysis of variance for repeated measurements, and P < 0.01 between the OVX group (or OVX + Roxadustat group) and the sham group in the following multiple comparisons among the three groups. Week 0: the start time of ovariectomy; Week 1: the start time of roxadustat administration. [file 13018_2022_3162_MOESM1_ESM.pdf]

Supplementary information

Roxadustat promotes osteoblast differentiation and prevents estrogen deficiency-induced bone loss by stabilizing HIF-1α and activating Wnt/β-catenin signaling pathway

Supplementary Table 1. The primer sequences for real-time PCR

Gene	Forward(5'-3')	Reverse(5'-3')
Runx2	AAATTAACGCCAGTCGGAGC	AGGACCGGGAACCACTTCTC
Sp7	AGGCCTTTGCCAGTGCCTA	GCCAGATGGAAGCTGTGAAGA
Alpl	GCAGTATGAATTGAATCGGAACAAC	ATGGCCTGGTCCATCTCCAC
Bglap	TTCTGCTCACTCTGCTGACC	TGCTTGGACATGAAGGCTTTG
Vegfa	GGAGTCTGTGCTCTGGGATTT	CCGTTGGCACGATTTAAGAGG
Glut1	AGCAGAGGCTTGCTTGTAGAG	ACGGACGCGCTGTAACTATG
Pdk1	GCAGACAGCTTACCTGAATCCT	CAGTACAACCATGTATGCTCAGT
Lef1	CAGCGCGAGACAATTATGGC	AGCTTCTCTTACCACCTGAAGT
Myc	TCAGCAACAACCGCAAGTG	CGTTCCAAGACGTTGTGTGT
Ccnd1	TGCGTGCAGAAGGAGATTGT	ACCTCCTCTTCGCACTTCTG
Mmp7	GCAGAATGTTCCTGGTTAGAGAAG	TCCTTGAGGTTGTCCACTAGACTAT
Hif1α	GAATGAAGTGCACCCTAACAAG	GAGGAATGGGTTCACAAATCAG
Actb	GCAAGCAGGAGTACGATGAGT	AAACGCAGCTCAGTAACAGTC

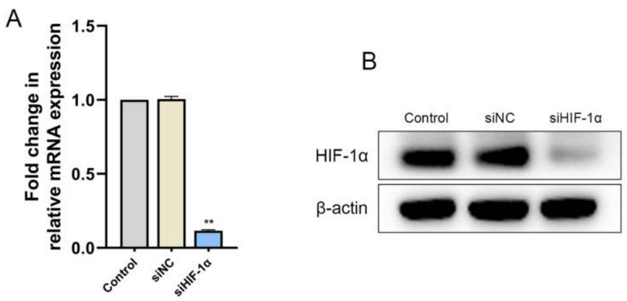

Supplementary Figure 1. The knockdown efficiency of siRNA against HIF-1α at the mRNA and protein levels in osteoblasts. (A) The mRNA expression of HIF-1α 24h after transfection. (B) The protein expression of HIF-1α 48h after transfection. Data are presented as the mean±SD of three independent experiments; **P<0.01, compared with the siNC group.

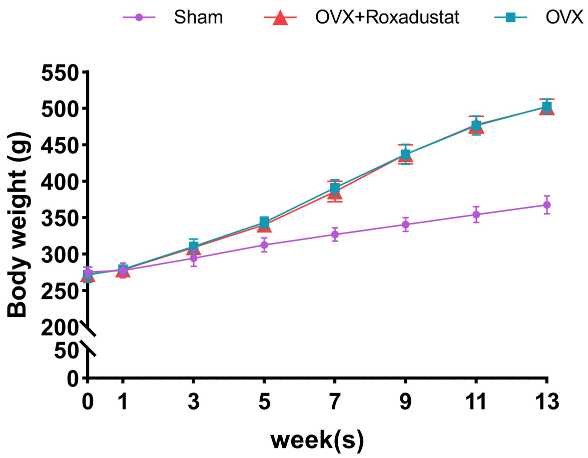

Supplementary Figure 2. Body weight changes during the 13-week experimental period in the Sham, OVX and OVX+Roxadustat groups. From week 3 to week 13, for each time point, P＜0.01 among the three groups in the analysis of variance for repeated measurements, and P＜0.01 between the OVX group (or OVX+Roxadustat group) and the sham group in the following multiple comparisons among the three groups. Week 0: the start time of ovariectomy; Week 1: the start time of roxadustat administration.
